# PC2 Ovotransferrin: Characterization and Alternative Immunotherapeutic Activity

**DOI:** 10.1155/2017/8671271

**Published:** 2017-03-20

**Authors:** Constantin Chiurciu, Viorica Chiurciu, Mariana Oporanu, Ionel Victor Pătrașcu, Iuliana Mihai, Mădălina Tablică, Romeo Teodor Cristina

**Affiliations:** ^1^S.C. Romvac Company S.A., Soseaua Centurii 7, Voluntari, 77190 Ilfov, Romania; ^2^Department of Pharmacology and Pharmacy, Faculty of Veterinary Medicine, Banat's University of Agriculture and Veterinary Medicine “King Michael I of Romania” from Timișoara, 119 Calea Aradului, 300645 Timișoara, Romania

## Abstract

Characterization and evaluation of immunotherapeutic potential of ovotransferrin PC2 (OTf PC2) were performed in this study. The ovoprotein was obtained from egg white from hens immunized with bacterial antigens, pathogenic for humans. For the negative control samples, OTf was extracted from eggs collected from Specific Pathogen-Free (SPF) hens and purified by affinity chromatography on Protein G-agarose column with two eluting peaks: I, representing ovalbumin, and II, ovotransferrin. The final* apo-*OTf form was reached by successive precipitation with ammonium sulfate and citric acid and the* holo-*OTf form by saturating the* apo-*form with FeCl_3_. Multiple OTf PC2 samples were analyzed through Sodium Dodecyl Sulfate-Polyacrylamide Gel Electrophoresis (SDS-PAGE) and, based on the molecular marker migration model, the ovotransferrin (76.5 kDa) and ovalbumin (45 kDa) were detected. The agglutination reaction exhibited statistically significant high specificity of the multiple OTf PC2, by reacting with the antigens used for hens' immunization. Following ELISA, it was established that OTf PC2 from hyperimmune eggs has specificity for all antigens; the antibody titer was high, indicating that OTf PC2 possesses immunological properties similar to immunoglobulin Y (Ig Y). This study suggests that OTf PC2 immunological activity may play a crucial role in the prevention and treatment of infections resistant to antibiotics and OTf PC2 can also act as a valuable nutraceutical.

## 1. Introduction

Ovotransferrin (OTf; conalbumin) belongs to the family of transferrins. This group of bilobate proteins plays an essential role in binding ferric iron and retaining it in solution. OTf is a monomeric glycoprotein that has been isolated from egg white (albumen). It contains 15 disulfide bridges and has a molecular weight of 76.5 kDa and an isoelectric point of 6.1 [1, 2]. Structurally, OTf is the second major protein present in the egg white (12-13%) and it binds iron ions (Fe^3+^) in combination with an anion (usually bicarbonate) [3].

OTf is synthesized in the chicken oviduct by transferrin avian gene and deposited in the albumen, where it folds in the form of two globular lobes, each containing a coupling locus for the iron ions [1, 2, 4].

OTf appears in two forms:* apo-form* and* holo-*form. The* apo-*OTf form does not contain iron and can be destroyed by the physical and chemical treatments, while the* holo*-form fortifies iron and is steady in the process of proteolytic hydrolysis and heat denaturation [2, 5].

OTf can transport iron ions to the developing embryos and is an essential component of the egg's antimicrobial defense system. In the state of being harmed by bacterial populations, OTf adjusts the levels of iron ions in the body [6].

The antimicrobial properties of OTf are the consequence of its ability to sequester iron needed for the development of microorganisms [7, 8]. Until now, the antibacterial [9, 10], antiviral [11], growth inhibitor [12], antihypertensive [13], immune modulatory [14, 15], antitumoral, nutraceutical [16–18], or as a targeting molecule [19] activity of the OTf, or its derived peptides, has been validated. Recently, an antimicrobial peptide, OTAP-92, located in the OTF's N lobe, has been isolated. This peptide has proven to be an intense bactericidal activity on Gram-positive (e.g.,* Staphylococcus aureus*) and Gram-negative bacteria (e.g.,* Escherichia coli*) [20, 21]. It has also been recently discovered that OTf PC2 extracted from hyperimmune eggs exerts evident immunological properties [22, 23].

OTf is also used as an antiviral and antimicrobial preservative, substituting lactoferrin in many applications. Structure and function of OTf and serum transferrin are similar to the lactoferrin from milk. It has been demonstrated that addition of OTf to cow's milk (which is low in transferrin) significantly improved its antibacterial properties and made it comparable to human milk [24, 25].

The powders, granules, and other products obtained from the hyperimmune eggs have now applications in the food and nutraceutical industry and in pharmaceutics and cosmetics. OTf is successfully used as a valuable constituent of diverse products, such as the following: supplements fortified with iron, instant drinks, and protein bars for athletes, and it has an undeniable influence on health and performance [26].

In this study, we assessed the immunotherapeutic potential of ovotransferrin PC2 (OTf PC2). In 2014, our team found that OTf extracted from hyperimmune eggs exerted some specific precise immunological properties. OTf had the ability to react with the particular epitopes of bacteria, viruses, and fungi that the chickens were immunized with.

## 2. Material and Methods

### 2.1. Animals

The study was conducted in the Laboratory for Research and Development of Romvac Company SA, Romania, and lasted six weeks, during which three inoculations and three replications were performed. All animals in the experiment (experimental and SPF animals) were provided by Romvac Company SA. Healthy Rhode Island Red hens were used in this study (2.5 ± 0.1 kg), with seven experimental groups of 20 individuals/group, at the age of 23 weeks and with an organized laying onset. The hens were housed in hall number 2 research facility at Romvac Company, in batteries, where the environmental temperature was maintained at 20 ± 2°C with relative humidity at 55 ± 10%. The fodder was ad libitum with standard diet R 21.5 for poultry. During the investigation period, the light cycle was as follows: 12 hours' light/dark period and the hens were kept in a quiet environment. Also, two groups of adult rabbits (10 individuals/group), average weight of 2 kg (2.0 ± 0.1 kg), were used in this experiment. They were kept in cages with 2 rabbits/cage, in the same housing conditions like that for the hens. Before the start of the experiment, poultry and rabbits were maintained for one week to acclimatize. All the procedures were carried out in accordance with the Directive 2010/63/EU on handling of animals used for scientific purposes and guidelines of the National Research Council (NRC), Institute of Laboratory Animal Research [27, 28]. The experiment was approved by the Ethical Committee of the Romvac Company SA, Romania.

### 2.2. Antigens

Strains used in this study were obtained from the Infectious and Tropical Diseases Hospital “*Victor Babes,*” Bucharest, and they were as follows:* Streptococcus pneumoniae, Staphylococcus aureus, Klebsiella pneumoniae, Acinetobacter baumannii, Escherichia coli* (with antigens grown in nutrient broth, Oxoid) and, respectively, from Romvac Company's microbiology lab collection:* Streptococcus mutans* (ATCC 55670),* Salmonella typhimurium* (RO 05 TL3/2014),* Salmonella enteritidis* (TL 248), and* Helicobacter pylori* (ATCC 49503).* Helicobacter pylori* was cultured in BHI medium (Bacto) and* Candida albicans* in Sabouraud liquid medium (HiMedia).* Clostridium difficile* was grown in thioglycollate medium (Oxoid). Cultures were incubated at 37°C, washed twice with sterile PBS at pH 7.2, and inactivated with 0.5% formaldehyde for 18 hours, after which the suspensions were adjusted to 0.05 at OD_600_, corresponding to a cell density of approximately 1 × 10^5^ CFU/mL.

### 2.3. Hens' Immunization

Hens were inoculated at the beginning of laying period, by i.m. way, three times, with the 12 prepared (multiple) antigens, representing 20,0 *μ*g protein/mL, from each studied bacterial strain suspended in sterile PBS (pH = 7.2 ± 0.2). The mixture was emulsified in an equal volume of adjuvant QS-21 (Natural Response SA, Chile). The applications were performed by injections in four different points on the breast muscles (0.25 mL/one point). The inoculation was repeated at 14 days and, respectively, at 4 weeks after the primary inoculation. Hyperimmune eggs were collected daily, after two weeks of the last inoculation, and were kept at 4°C for the albumen processing. For the control group, Specific Pathogen-Free (SPF) 30-week-old hens were used, obtained from Romvac Company. Eggs from these hens were used as the negative control samples in immunoassay performed.

### 2.4. Ovotransferrin Separation

OTf PC2 was obtained from hyperimmune eggs (HPC2) from chickens immunized with bacterial and fungal antigens (multiple antigens). Ovotransferrin can be separated from the albumen through several methods, such as the following: ethanol fractionation [29] and ammonium sulfate precipitation or by ovalbumin coagulation [30, 31], isolated by different types of chromatography [32, 33]. Fractions from albumen obtained on DEAE Affi-Gel Blue columns are further purified by liquid chromatography using Q Sepharose Fast Flow methodology [34]. In the study performed by Ko and Ahn, OTf was obtained by diluting the albumen twice with deionized water and, to prevent distortion during the separation process, the* apo*-form was converted into the* holo* one, by adding the solution of FeCl_3_  ×  6H_2_O. OTf with iron was obtained by using various concentrations of ethanol at pH 9 [35]. The resulting precipitate was dissolved in deionized water and iron was removed by passing through the ion exchange using resin AG_1_-X_2_ [36, 37].

The* apo-* and* holo-*OTf PC2 and, respectively, OTf SPF samples were separated by precipitation and purification techniques. OTf purification was performed by* affinity chromatography on Protein G-agarose column* (Thermo Scientific), and the fractions purity testing was accomplished by SDS-PAGE.

### 2.5. Preparation of* Apo*-Form Ovotransferrin (Apo-OTf)

The* apo-*OTf was prepared following the adaptation of the method described by Abeyrathne et al. [30]. The albumen separated from the yolk was diluted with deionized water in the ratio 1 : 1 and then homogenized. The pH values were adjusted to 4.5–5.0 and the suspension was kept overnight at 4°C. Later, ovomucin was removed and the resulting suspension was precipitated with ammonium sulfate 5% (w/v) and 2.5% (w/v) citric acid and then centrifuged at 3400 ×g for 40 minutes at 4°C. The supernatant was removed and the precipitate containing ovotransferrin was dissolved in two volumes of deionized water. Next, the deposit was reprecipitated with 2.5% (w/v) ammonium sulfate and 1.5% (w/v) citric acid and, after centrifugation, the precipitate was filtered in two volumes of deionized water, to remove all salts. The* apo-*OTf purity was determined by SDS-PAGE (Sodium Dodecyl Sulfate-Polyacrylamide Gel Electrophoresis).

### 2.6. Preparation of Holo-Form Ovotransferrin (Holo-OTf)

This was performed as described by Ko and Ahn [35]. The albumen diluted in the* ratio* 1 : 1 with deionized water was adjusted to pH 9.0 and a solution of NaHCO_3_ and FeCl_3_  × 6H_2_O, 0.5 M, was added to reach the concentration of 20 mM. Next, four volumes of cold 100% ethanol (to reach a final concentration of 43%) were added to the albumen-iron suspension. The* holo-*OTf egg white was separated by centrifugation at 3200 ×g for 40 minutes. The supernatant fluid (S_1_) was removed and saved, and the deposit was reprecipitated with 43% ethanol and was recentrifuged again at 3200 ×g for another 40 minutes. The second supernatant fluid (S_2_) was mixed with the first one (S_1_) and, after filtration, cold ethanol (supernatant : ethanol = 5 : 2) was added, to reach the final concentration of 59%. The* holo*-OTf containing precipitate was dissolved in nine volumes of deionized water, ethanol was removed from suspension by ultrafiltration, and pH was adjusted to 4.7 using 0.5 M citric acid. Iron removal was accomplished using ion exchange resin AG_1_-X_2_ (Bio-Rad).

### 2.7. Preparation of the Anti-OTf Rabbit Sera

OTf anti-sera were prepared by rabbits' hyperimmunization with OTf SPF. The OTf in the concentration of 5 mg/mL protein was emulsified in Montanide ISA 70 adjuvant (SEPPIC). First inoculation was done by intradermal (i.d.) route with dose of 2 mL antigen administered to 3-4 points on both sides of the body. Second inoculation was performed at 21 days after the first administration, in the same location and with the same dose and the same way of administration as previously. Third inoculation was done 14 days after the second inoculation and blood was collected 7 days after the last inoculation. Rabbit anti-OTf IgG was obtained using precipitation technique and ion exchange chromatography. The total OTf protein concentration (mg/mL) was assessed by the Bradford method.

### 2.8. Sodium Dodecyl Sulfate-Polyacrylamide Gel Electrophoresis (SDS-PAGE)

Electrophoresis was performed using the general principle [38] and Omni Page electroblotter (Cleaver Scientific). OTf PC2 of the test samples was diluted to a final concentration of 2 mg/mL protein, using 2-mercaptoethanol Laemmli buffer (Sigma Aldrich) and bromophenol blue (Sigma Aldrich). After incubating the samples for 10 minutes at 96°C, 5 *μ*L of each sample was loaded on a polyacrylamide migration gel of 10% and a concentration gel of 4%. A molecular marker, Protein Marker VI (AppliChem), containing a mixture of 12 proteins with molecular weight ranging from 10 to 245 kDa, was also run on the gel. Electrophoresis was performed at 90 mV and 185 mA, for 90 minutes, and staining was performed with Coommassie Brilliant Blue R250 (Sigma Aldrich).

### 2.9. ELISA Immunoassay Test

For OTfPC2, identification and quantification were performed on a SpectraMax 190 Microplate Reader (Molecular Devices) and standardized kits were used (MyBioSource). An “*in-house*” ELISA was applied to assess the specificity of OTf PC2 to bacterial antigens. To achieve this, 96-well microplate (Greiner Bio-One) was coated with each antigen, in part inactivated, at a protein concentration of 10 mg/mL in carbonate-bicarbonate buffer (0.05 M, pH 9.6). After 12 hours of incubation at 37°C the micro plates were washed with PBS-Tween. Blocking of nonspecific adsorption was done with 1% BSA solution (Merck) for 30 minutes. Samples, diluted in PBS buffer at pH 7.4, were distributed evenly in the wells, together with the positive and negative controls; wells A1 and H1 were left as blank. Plate was incubated for 2 hours at 37°C. After washing with PBS-Tween, the horseradish peroxidase- (HRP-) labeled goat anti-OTf IgG (detection antibody, MyBioSource) was added to each well. Plate was read at a wavelength of 450 nm, after the addition of TMB chromogenic substrate (SurModics), and the reaction was stopped with 1 N HCl.

### 2.10. Double-Sandwich ELISA

Quantification of OTf by this test was performed using Chicken Ovotransferrin ELISA Kit (MyBioSource); 100 *μ*L of serial dilutions of standard OTf in PBS was distributed in each well (6.75 ng/mL, 12.5 ng/mL, 25 ng/mL, 50 ng/mL, 100 ng/mL, and, resp., 200 ng/mL) and OTf samples were analyzed. The plate was incubated in dark place for 30 minutes at 25°C, covered. After incubation the plate was washed 4 times with washing buffer (Wash Buffer) diluted 20 times. To each well 100 *μ*L of the peroxidase (HRP) labeled enzyme conjugate was added, diluted 100 times. The plate was incubated in the dark for 20 minutes at 24°C and to each well 100 mL of TMB was added and kept at the room temperature for 10 minutes. Next, 100 *μ*L/well of stop solution was added and absorbance was read immediately at 450 nm (OD 450) using a SpectraMax 190 Absorbance Microplate Reader (Molecular Devices). OTf purified in serial dilutions of PBS was used to create a standard curve. This curve was used to estimate the OTf's total concentration in the samples.

### 2.11. Rapid Agglutination Reaction (RAR)

It was performed on glass plates with the wells (Dacchim). To obtain the reaction, inactivated bacterial antigens and OTf PC2 samples were used. OTf SPF was used as a negative control reaction.

Ten* apo-*OTf PC2 series were isolated from hyperimmune egg albumen by double precipitation with ammonium sulfate (5% and 2.5%) and citric acid (2.5% and 1.5%) and 5* holo-*OTf series, by precipitation with ethanol of 43% and 59%. Iron coupling was achieved with 0.5 M FeCl_3_  × 6H_2_O solution in the presence of bicarbonate anion. The removal of Fe (3+) was accomplished by ion exchange AG_1_-X_2_ resin using 0.9 g per 100 mL* holo-*OTf. The obtained* apo-*OTf was pale-white and the* holo-*OTf was brick-red.

Multiple OTf PC2 was characterized by the following:* Agar Gel Immunodiffusion* (AGID),* Immunodiffusion Simple Radial Test* (IDSR),* Rapid Agglutination Reaction* (RAR), and ELISA “*in-house*” and “*double-sandwich*” tests.

*Agar Gel Immunodiffusion* (AGID) was performed on 1% Noble Agar (Difco) prepared in borate buffer at pH 8.6. The mixture was boiled in the water bath until agar completely dissolved; 17 mL of warm agar at 45–60°C was poured into Petri dishes with a diameter of 90 mm. Seven wells were made with a gel stencil (one central and six at equal distances around) with a diameter of 6 mm and 3 mm distance between them. To establish the identity, in the wells 3 and 5, OTf international standard (MyBioSource) was deposited; in wells 2 and 6, test OTf was placed and in wells 1 and 4 and central well rabbit IgG anti-OTf. The identity establishing between* holo*-OTf and* apo-*OTf occurred by their repartition in wells 3 and 5 and, respectively, in wells 2 and 6, the reaction volume was 40 *μ*L/each well. Next, binary dilutions of OTf samples were made (from 1/2 to 1/32) in order to establish the optimal working dilutions for the OTf* apo-* and* holo*-forms. Precipitation reactions were read after 24 hours. In case of positive reaction, an OTf standard line joins the line on OTf sample analyzed and is situated midway between the wells. Weak positive line is not continuous being tangent to the well of the analyzing sample.

*Immunodiffusion Simple Radial Test* (IDSR) was used to determine the OTf concentration. It was performed in 2% agarose Type II gel, medium grade (Sigma Aldrich), prepared in borate buffer at pH 8.6. After boiling, the solution was kept in a water bath at 56°C; the OTf antiserum was diluted separately in the same buffer and kept in a water bath at the same temperature for 15 minutes. Equal amounts of gel were mixed with antisera depending on the titer and poured into Petri dishes with a diameter of 50 mm. After agarose solidification, 3 mm diameter wells were made with a stencil, one central and five peripheral. In the central well, 20 *μ*L of standard OTf (MyBioSource) was added, and, in lateral wells, OTf samples were deposited. After 24 hours, the precipitation zone diameters were measured using a caliper (Digital Caliper SAE-Metric and Digital Display) and the OTf concentration values (mg/mL) were read on a standard curve.

### 2.12. Statistical Analysis

The statistical analysis for OTf was conducted using Wilcoxon Signed-Rank Test, operating statistical software package GraphPad Prism 5.0 for Windows (GraphPad Software, San Diego, USA). The Wilcoxon Signed-Rank Test is the nonparametric test equivalent to the dependent* t*-test, with statistical significance reached at *P* < 0.05 or lower.

## 3. Results and Discussions

**Figure 1:**
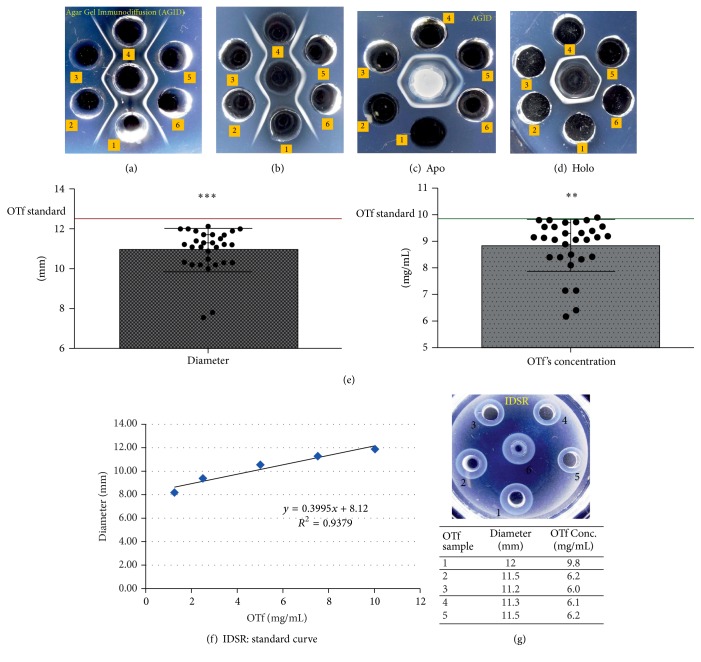
Analyzing the OTf values. (a) Agar Gel Immunodiffusion (AGID) Test: the identity establishing between standard OTf (wells 3 and 5) and* apo-*OTf PC2 (wells 2 and 6); wells 1 and 4 and central well are anti-OTf rabbit IgG. (b) AGID: the identity between* holo-*OTf (wells 3 and 5) and* apo-*OTf PC2 (wells 2 and 6); wells 1 and 4 and central well are anti-OTf rabbit IgG. (c) AGID: test for* apo-*OTf (full OTf and dilutions from 1/2 to 1/32). (d) AGID: test for* holo-*OTf (full OTf and dilutions from 1/2 to 1/32). (e) The statistical analysis of OTf concentration values (mg/mL) obtained by IDSR test according to the diameter of the precipitation rings (mm) and concentration. The values *P* < 0.0001^*∗∗∗*^ and *P* = 0.003^*∗∗*^ are indicating a high statistical significance. (f) The standard curve for quantitative determination of OTf by IDSR. (g) Quantification of OTf samples by IDSR: central well: the standard OTf; wells 1 and 2:* holo-*OTf; wells 3, 4, and 5:* apo-*OTf and the OTf's concentration values (mg/mL), depending on the precipitation ring diameters (mm).

Multiple OTf PC2 against standard OTf (MyBioSource) was comparatively tested by AGID. Our results indicated the similarity between the standard OTf (wells 3 and 5) and the multiple PC2 OTf (wells 2 and 6) against the rabbit anti-OTf IgG. The precipitation line presented continuity between the two compared samples (Figure 1(a)).

The reaction of identity between the two forms of OTf PC2 was also highlighted (Figure 1(b)). The* apo-* and* holo*-OTf forms were tested in dilutions extending from 1/2 to 1/2048 by AGID against OTf IgG. For the* apo-*OTf form, the optimal dilution established was 1/8 and, for* holo-*OTf form, the optimal dilution was 1/16 (Figures 1(c) and 1(d)).

To quantify the OTf from samples, the IDSR test was used. OTf concentration values (mg/mL) obtained by IDSR according to the diameter of the precipitation rings (mm) were analyzed statistically and revealed highly significant values (Figure 1(e)).

The OTf concentration was read on a standard curve. The calibration curve for this method was achieved by plotting the OTf' standard concentration (mg/mL) correlated with the diameter of the precipitation rings measured in millimeters (mm). Data indicated that the OTf PC2 concentration values have varied and were between 6.1 and 9.8 mg/mL depending on the size of the precipitation rings (Figures 1(f) and 1(g)).

**Figure 2:**
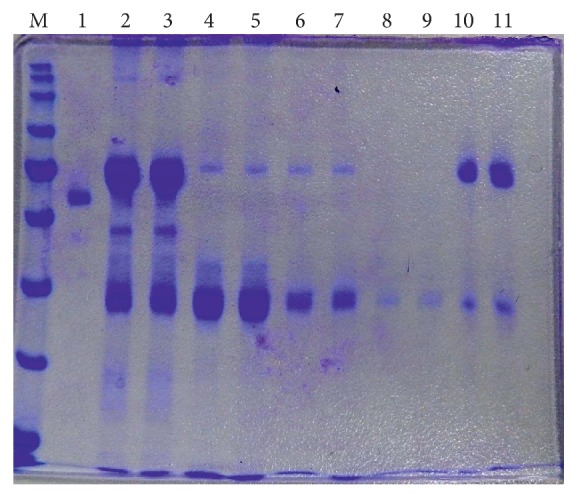
Testing of* apo-* and* holo*-OTf by SDS-PAGE: 0: protein marker (M), 1: OTf Standard ELISA Kit, 1/100 dilution; 2 and 3:* apo*-OTF multiple sample, 1/25 dilution; 4 and 5:* apo*-OTF multiple sample, 1/30 dilution; 6 and 7:* holo*-OTf multiple sample, 1/15 dilution; 8 and 9:* holo*-OTf multiple sample, 1/30 dilution; 10 and 11:* holo*-OTf sample Fe, decoupling 1/1 dilution.

Samples of* apo-* and* holo-*OTf PC2 were also analyzed by electrophoresis in polyacrylamide gel under denaturing conditions. This was done to identify the presence of* apo-* and* holo-*OTf PC2 and to assess their purity. Based on the molecular marker migration model, two protein fractions were identified: ovotransferrin (OTf) with 76.5 kDa molecular weight and ovalbumin (OVA) with 45 kDa (Figure 2).

The quantification revealed that the molecular weights of both,* apo-* and* holo*-forms, are the same, namely, 76.5 kDa. It was also noted that the removal of iron from the* holo*-form, by using ion exchange chromatography resin AG_1_-X_2_ does not alter the OTf's immunological properties. The* apo*-OTf purification was performed by means of the affinity chromatography on Protein G-agarose column. Two peaks of elution were obtained by reading fractions at 280 nm: peak I (fractions 3 and 4 with registered values between 0.163 and 0.278 nm) and peak II (fractions 7 and 8 with values comprised between 0.275 and 0.469 nm).

**Figure 3:**
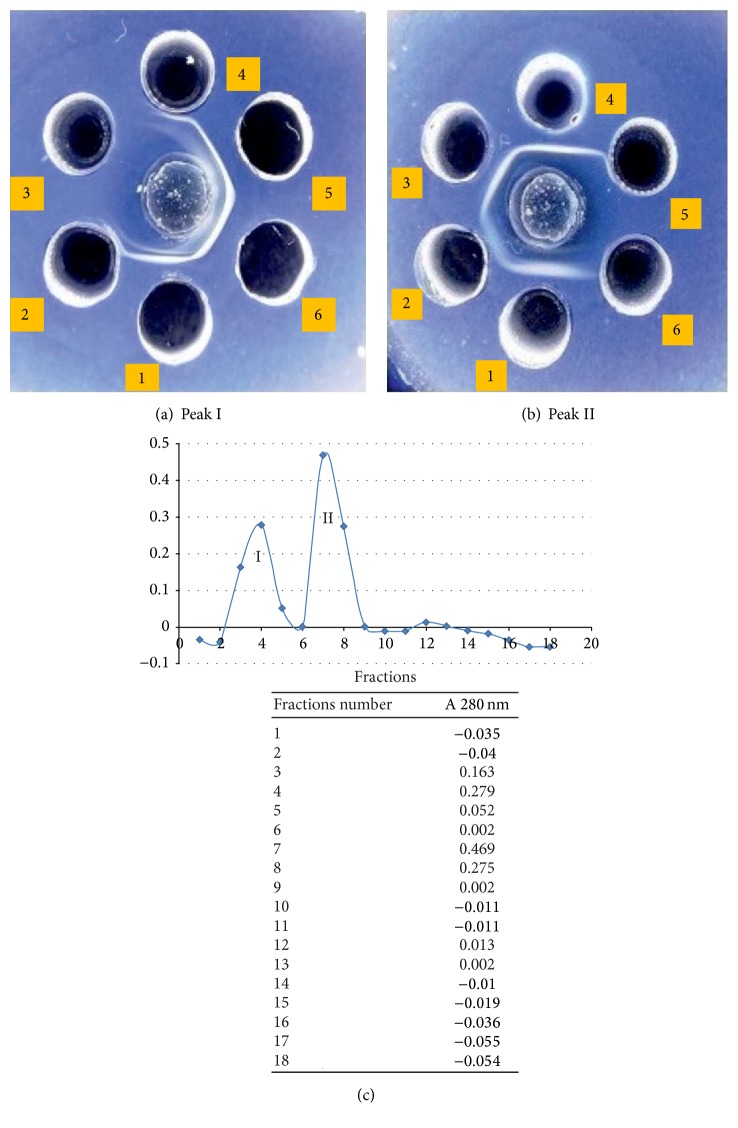
AGID testing of the fractions obtained from ovotransferrin by affinity chromatography with Protein G-agarose column: (a) (Peak I) central well: rabbit IgG anti-OVA; well 1: OVA standard wells 2, 3, 4, 5, and 6; (b) (Peak II) central well: rabbit IgG anti-OTf; wells 1, 2, 3, 4, 5, and 6. (c) The peaks of the elution profile obtained after affinity chromatography on Protein G-agarose column: two peaks of elution were obtained by reading fractions at 280 nm: peak I (fractions 3 and 4 with registered values between 0.163 and 0.278 nm) and the fractions of peak II (fractions 7 and 8 with values comprised between 0.275 and 0.469 nm).

Testing of peak I fractions against rabbit anti-ovalbumin IgG revealed the ovalbumin's (OVA) presence. The peaks of the elution profile obtained are shown in Figure 3.

**Figure 4:**
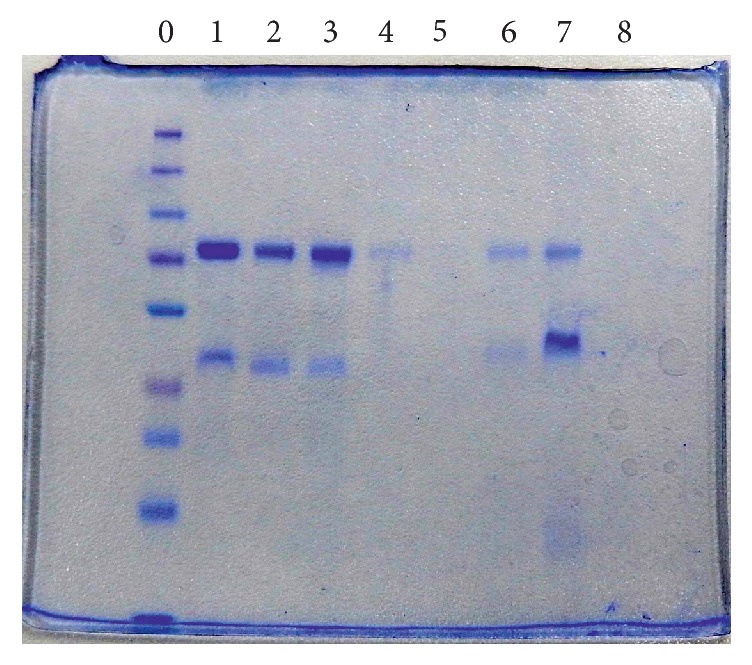
SDS-PAGE purity analysis of obtained OTf fractions by column chromatography with Protein G-agarose: 0: protein marker; 1: full OTf; 2: fraction 4; 3: fraction 5; 4: fraction 6; 5: fraction 7; 6: fraction 8; 7: fraction 9; 8: fraction 10.

It was observed that each fraction is represented by a single clear line of precipitation located halfway between wells. Fractions 6 and 7 showed a single band with a molecular mass of 76.5 kDa, which demonstrated the OTf's purity, as assessed by SDS-PAGE (Figure 4).

**Figure 5:**
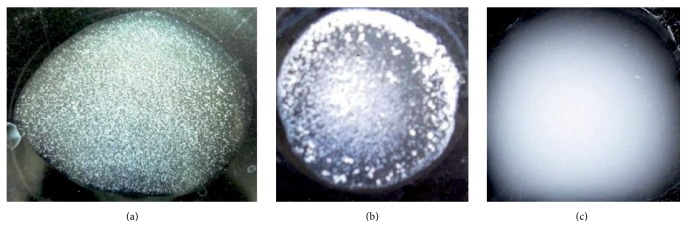
*Rapid Agglutination Reaction.* (a) presence of small granular particles between* apo-*OTf multiple antigen dilution of 1/16 and* Salmonella *sp. (b) Agglutination with large particles, having the floaters appearance, between* holo-*OTf multiple antigen dilution of 1/16 and* Salmonella *sp. (c) Case of OTf SPF negative reaction and the same antigen: the suspension maintained its homogeneity.

The specificity of* apo-* and* holo-*forms of OTf PC2 has been expressed by Rapid Agglutination Reaction. Agglutination revealed the presence of small granular particles between* apo-*OTf multiple antigen dilution of 1/16 and* Salmonella *sp. The agglutination with large particles, with floaters appearance, between* holo-*OTf multiple antigen dilution of 1/16 and* Salmonella *sp. was also detected. In the case of OTf SPF negative reaction and the alike antigen, the suspension maintained its homogeneity (Figure 5).

The specificity of multiple OTf PC2 compared with multiple IgY against antigens used for immunization, tested by RAR, is shown in Table 1.

**Figure 6:**
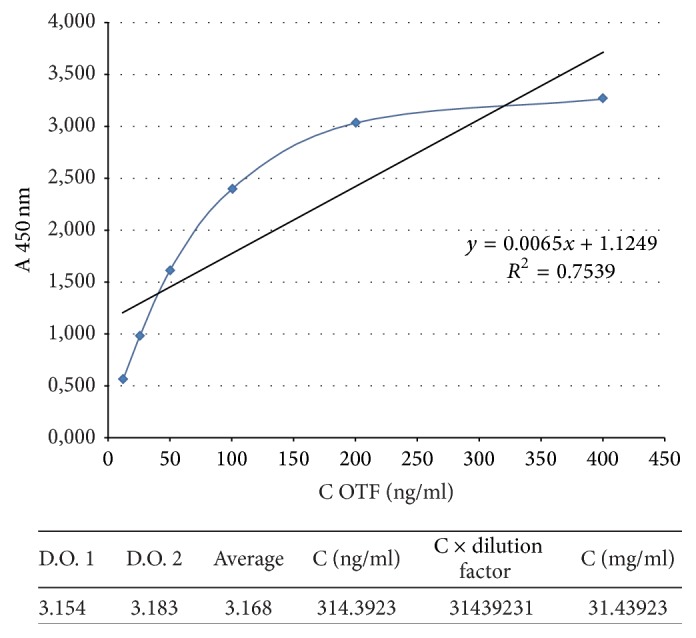
The calibration curve for the OTf quantitative determination by “*sandwich*” ELISA method. Values measured for OTf: standard calibration curve was performed, in which the equation was as follows: OD 450 nm = 0.0065*x* + 1.1249.

Data indicates that OTf SPF had no immunological properties and did not react with the bacterial antigens; however, the OTf showed similar immunological character as the immunoglobulin Y (IgY) present in the egg yolk. PC2 OTf quantitative determination was accomplished by ELISA “*sandwich*” method. Based on absorbance values measured for OTf, standard calibration curve was performed, in which the equation was as follows: OD 450 nm = 0.0065*x* + 1.1249. Applying this equation, the calculation of OTf concentration from samples was performed (Figure 6).

**Figure 7:**
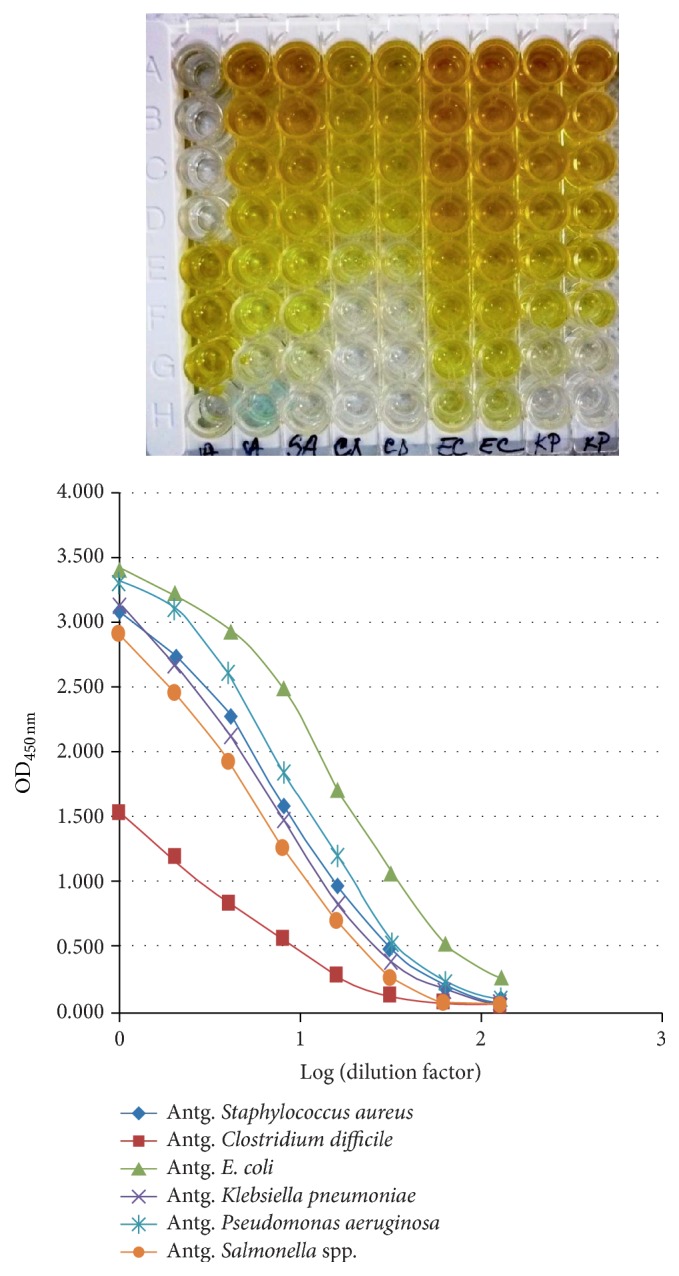
Testing of the OTf PC2 specificity by ELISA against some bacterial antigens:* Staphylococcus aureus* (SA),* Clostridium difficile* (CD),* Escherichia coli* (EC), and* Klebsiella pneumoniae* (KP) antigens.

To demonstrate the specificity between OTf PC2 and the bacterial antigens, used to immunize hens, direct ELISA test was applied. From multiple OTf PC2 samples, binary dilutions were made, starting with 1 : 100. The results obtained and shown in Figure 7 revealed that OTf extracted from hyperimmune egg white has a high specificity for all bacterial antigens. The titer of specific antibodies is high but different for each antigen separately (e.g., OD = 3.400 for* Escherichia coli*; OD = 3.200 for* Pseudomonas aeruginosa*; OD = 3.100 for* Staphylococcus aureus*; OD = 3.100 for* Klebsiella pneumoniae*; OD = 2.900 for* Salmonella *sp.; OD = 1.500 for* Clostridium difficile*). This demonstrates that the hen's immune system responds equally to antigenic stimuli inoculated.

Our results show that OTf can be isolated from egg albumen using two precipitation methods and this broadens its possibility of use in various fields of research. The results presented here are consistent with the data published by Abeyrathne et al. [30] and Ko and Ahn [35] in relation to ovotransferrin obtaining and characterization. The hyperimmune egg PC2 differs from the consumption one, due to its abundant content of specific antibodies, immune-modulators, transfer factors, and peptides.

Our work is in consonance with the results of Azari and Baugh [29] and Evans et al. [36], who have demonstrated the antibacterial activity of* holo-*OTf, capable of sequestering iron required for the growth of microorganisms. Therefore, the addition of iron is an important step in ovotransferrin purification in all cases, as other authors stated [35]. From the PC2 hyperimmune egg albumen, we isolated ovotransferrin (OTf-PC2), which exerted certain specific ability to react with bacteria, viruses, and/or fungi epitopes and these properties have been previously studied by us [22, 23].

The identity between multiple OTf PC2 and OTf international standard was established by AGID test. We observed that both* apo-* and* holo-*forms retained the immunological property of reacting with anti-OTf rabbit IgG, resulting in a clear precipitation line located midway between the wells. The IDSR test allowed quantitative determination of multiple OTf PC2, and values obtained were between 6.1 and 9.8 mg/mL.

The SDS-PAGE assay performed in our study confirmed that the molecular weight of OTf PC2 (*apo* and* holo*) is 76.5 kDa. Purification of multiple OTf PC2 by elution on Protein G-agarose column showed two peaks (the fractions belonging to peak II were represented by the purified OTf). The RAR test revealed the OTf PC2 (*apo* and* holo*) specificity, by reacting with bacterial antigens used in immunization. In contrast, we determined that OTf SPF did not have this immunological property, with the obtained reactions being negative.

## 4. Conclusion

The PC2 OTf specificity study proves an important vector role for this protein. This is specifically exhibited in reacting with antigens' epitopes used to immunize hens and revealing certain immunological properties of PC2 OTf isolated from the hyperimmune eggs. Here, we have demonstrated that multiple PC2 OTf extracted from the hyperimmune eggs showed specificity towards all bacterial antigens used to inoculate hens in our experiment.

The OTf PC2 immunological activity demonstrated in this study may be used with certainty as alternative biological means in the prevention and treatment of antibiotic resistant infections. OTf can be used as a medicine, meaning to achieve therapeutic effects in humans and animals. Also, the use of OTf PC2 as nutraceuticals could be a gain in the biomedical investigation due to their role as natural means for prevention of diverse health issues. The outcome of this research could be considered as an ab initio reference for other studies to come.

## Figures and Tables

**Table 1:** RAR-testing of multiple PC2 OTf specificity compared with multiple IgY against bacterial antigens used in the study.

Bacterial species/antigens	OTf PC2 multiple	OTf SPF	IgY multiple
*Staphylococcus aureus*	+	−	+
*Escherichia coli*	+	−	+
*Klebsiella pneumoniae*	+	−	+
*Candida albicans*	+	−	+
*Acinetobacter baumannii*	+	−	+
*Pseudomonas aeruginosa*	+	−	+
*Helicobacter pylori*	+	−	+
*Streptococcus mutans*	+	−	+
*Salmonella typhimurium*	+	−	+
*Salmonella enteritidis*	+	−	+
*Clostridium difficile,* anatoxin	+	−	+
*Clostridium difficile,* bacterial bodies	+	−	+

Total	12/12	12/12	12/12

## Abbreviations

OTf: Ovotransferrin
OTf PC2: Ovotransferrin Pătrașcu I.V. + Chiurciu C. + Chiurciu V.
SPF: Specific Pathogen-Free
ad libitum: At will
BHI: Brain-heart infusion
PBS: Phosphate Buffered Saline
CFU: Colony-forming unit
OD: Optical density
SDS-PAGE: Sodium Dodecyl Sulfate-Polyacrylamide Gel Electrophoresis
BSA: Bovine Serum Albumin
HRP: Horseradish Peroxidase
TMB: 3,3′,5,5′-TetraMethylBenzsidine
AGID: Agar Gel Immunodiffusion
IDSR: Immunodiffusion Simple Radial Test
RAR: Rapid Agglutination Reaction
OVA: Ovalbumin.

## References

[1] Warner R. C., Neurath H., Bailey K. (1954). Egg proteins. *The Proteins*.

[2] Thomas J. W., Elleman C., Kingston I. B., Wilkins A. G., Kuhn K. A. (1982). The primary structure of hen ovotransferrin. *European Journal of Biochemistry*.

[3] Battistuzzi G., Sola M. (1992). Fe3+ binding to ovotransferrin in the presence of *α*-amino acids. *Biochimica et Biophysica Acta (BBA)/Protein Structure and Molecular*.

[4] Patrascu I. V. Ovotransferina PC2: O formă nouă imunogenică activă la nivel molecular. *Curs de Medicina Integrativă cu tema Imunoglobulinele Moderne si Patologiile Actuale*.

[5] Castro M. M., Lopez-Alonso Fandiño R., Recio Sanchez M. I., Ramos Gonzalez M. M., De Artiñano A. A. Bioactive peptides derived from the proteins of egg white by means of enzymatic hydrolysis.

[6] Hiidenhovi J., Mäkinen J., Huopalahti R., Ryhänen E.-L. (2002). Comparison of different egg albumen fractions as sources of ovomucin. *Journal of Agricultural and Food Chemistry*.

[7] Oppenheimer S. J. (1989). Iron and infection: the clinical evidence. *Acta Paediatrica Scandinavica, Supplement*.

[8] Bullen J. J. (1981). The significance of iron in infection. *Reviews of Infectious Diseases*.

[9] Valenti P., Antonini G., von Hunolstein C., Visca P., Orsi N., Antonini E. (1983). Studies on the antimicrobial activity of ovotransferrin. *International Journal of Tissue Reactions*.

[10] Guo M., Harvey I., Yang W. (2003). Synergistic anion and metal binding to the ferric ion-binding protein from Neisseria gonorrhoeae. *Journal of Biological Chemistry*.

[11] Giansanti F., Massucci M. T., Giardi M. F. (2005). Antiviral activity of ovotransferrin derived peptides. *Biochemical and Biophysical Research Communications*.

[12] Topilescu G., Chiurciu V., Patrascu I. V., Chiurciu C., Cristina R. T. (2014). Growth Inhibition of antibiotic resistant bacteria by neutralizing IgY antibodies. *Journal of Biotechnology*.

[13] Miguel M., Aleixandre A. (2006). Antihypertensive peptides derived from egg proteins. *Journal of Nutrition*.

[14] Polanowski A., ZabŁocka A., Sosnowska A., Janusz M., Trziszka T. (2012). Immunomodulatory activity accompanying chicken egg yolk immunoglobulin Y. *Poultry Science*.

[15] Masona A., Stephen B., Brown A., Church W. R. (1988). Domain-specific monoclonal antibodies to ovotransferrin indicate conservation of determinants involved in avian transferrin receptor recognition. *Comparative Biochemistry and Physiology B: Comparative Biochemistry*.

[16] Abeyrathne E. D. N. S., Lee H. Y., Ahn D. U. (2013). Egg white proteins and their potential use in food processing or as nutraceutical and pharmaceutical agents—a review. *Poultry Science*.

[17] Giansanti F., Leboffe L., Angelucci F., Antonini G. (2015). The nutraceutical properties of ovotransferrin and its potential utilization as a functional food. *Nutrients*.

[18] Triszka T., Rozanski H., Polanovski A. (2013). Eggs as a very promising source of biomedical and nutraceutical preparations: a review. *Journal of Life Sciences*.

[19] Ibrahim H. R., Tatsumoto S., Hajime O., Van Immerseel F., Raspoet R., Miyata T. (2015). A novel antibiotic-delivery system by using ovotransferrin as targeting molecule. *European Journal of Pharmaceutical Sciences*.

[20] Ibrahim H. R., Iwamori E., Sugimoto Y., Aoki T. (1998). Identification of a distinct antibacterial domain within the N-lobe of ovotransferrin. *Biochimica et Biophysica Acta—Molecular Cell Research*.

[21] Ibrahim H. R., Sugimoto Y., Aoki T. (2000). Ovotransferrin antimicrobial peptide (OTAP-92) kills bacteria through a membrane damage mechanism. *Biochimica et Biophysica Acta—General Subjects*.

[22] Pătrașcu I. V., Chiurciu V., Chiurciu C., Oporanu M., Topilescu G., Mihai I. Manufacture and use of modern ovotransferrin (OTf-M).

[23] Pătrașcu I. V., Chiurciu V., Chiurciu C., Oporanu M., Topilescu G., Mihai I. Preparation and use of immunologically active ovotransferrins (OTf-PC-2).

[24] Giansanti F., Rossi P., Massucci M. T. (2002). Antiviral activity of ovotransferrin discloses an evolutionary strategy for the defensive activities of lactoferrin. *Biochemistry and Cell Biology*.

[25] Kovacs-Nolan J., Phillips M., Mine Y. (2005). Advances in the value of eggs and egg components for human health. *Journal of Agricultural and Food Chemistry*.

[26] Toldara F., Nollet L. M. L. (2013). *Proteomics in Food: Principles and Applications*.

[27] (2010). Directive 2010/63/EU of the European Parliament and the Council of 22 September 2010 on the protection of animals used for scientific purposes. *Official Journal of the European Union L*.

[28] National Research Council Institute of Laboratory Animal Research (NRC) (1996). *Guide for Care and Use of Laboratory Animals*.

[29] Azari P., Baugh R. F. (1967). A simple and rapid procedure for preparation of large quantities of pure ovotransferrin. *Archives of Biochemistry and Biophysics*.

[30] Abeyrathne E. D. N. S., Lee H. Y., Ham J. S., Ahn D. U. (2013). Separation of ovotransferrin from chicken egg white without using organic solvents. *Poultry Science*.

[31] Abeyrathne E. D. N. S., Lee H. Y., Ahn D. U. (2014). Sequential separation of lysozyme, ovomucin, ovotransferrin,and ovalbumin from egg white. *Poultry Science*.

[32] Awade A. C. (1996). On hen egg fractionation: applications of liquid chromatography to the isolation and the purification of hen egg white and egg yolk proteins. *Zeitschrift für Lebensmittel-Untersuchung und Forschung*.

[33] Al-Mashikhi S. A., Nakai S. (1987). Separation of ovotransferrin from egg white by immobilized metal affinity chromatography. *Agricultural and Biological Chemistry*.

[34] Vachier M. C., Piot M., Awadé A. C. (1995). Isolation of hen egg white lysozyme, ovotransferrin and ovalbumin, using a quaternary ammonium bound to a highly crosslinked agarose matrix. *Journal of Chromatography B: Biomedical Sciences and Applications*.

[35] Ko K. Y., Ahn D. U. (2008). An economic and simple purification procedure for the large-scale production of ovotransferrin from egg white. *Poultry Science*.

[36] Evans R. W., Kong X., Hider R. C. (2012). Iron mobilization from transferrin by therapeutic iron chelating agents. *Biochimica et Biophysica Acta. General Subjects*.

[37] Williams J., Evans R. W., Moreton K. (1978). The iron binding properties of hen ovotransferrin. *Biochemical Journal*.

[38] Creative BioMart (2015). *Principle and Protocol of Sodium Dodecyl Sulphate-Polyacrylamide Gel Electrophoresis (SDS-PAGE)*.

